# China's Low‐Carbon Energy Transition Over the Past Two Decades: Experiences and Implications

**DOI:** 10.1002/advs.77259

**Published:** 2026-08-19

**Authors:** Yu Liu, Zhiyu Tian, Zhengxian Zhang, Xinbei Li

**Affiliations:** ^1^ College of Urban and Environmental Sciences Peking University Beijing China; ^2^ Institute of Carbon Neutrality Peking University Beijing China; ^3^ Institute of Energy Environment and Economy Tsinghua University Beijing China; ^4^ Energy Research Institute National Development and Reform Commission Beijing China

**Keywords:** China, energy trilemma, low‐carbon energy transition, policy implications

## Abstract

With the rapid expansion of renewable energy, coal consumption in China briefly declined and then rebounded. Existing literature provides limited explanations for this dynamic pattern. This paper summarizes the experiences and implications of China's low‐carbon energy transition by adopting the energy trilemma as an analytical perspective and examining the corresponding policy instruments across different stages. The results show that the energy transition reflects a phased process of trade‐offs among economic development, energy security, and environmental sustainability. In the early stage, economic development was prioritized, with coal ensuring energy supply. As environmental constraints intensified, administrative measures were implemented to drive a temporary decline in coal consumption. Under the constraints of multiple objectives, China has adopted a gradual transition pathway that accelerates renewable energy development while facilitating the functional transformation of coal‐fired power rather than simply phasing it out. From a governance perspective, China advances the transition through a coordinated policy framework that sets long‐term direction by strategic planning, reinforces implementation with administrative measures, and gradually introduces market mechanisms to improve resource allocation. This study provides insights into China's low‐carbon energy transition and offers policy implications for other developing economies facing similar energy dilemmas.

## Graphical abstract

China's low‐carbon energy transition features a brief decline and subsequent rebound in coal consumption, alongside the rapid expansion of renewable energy. This study develops a stage‐based analytical framework to explain this evolution from the perspectives of economic development, energy security, and environmental sustainability, revealing how policy instruments manage trade‐offs among these objectives and offering insights for developing economies.

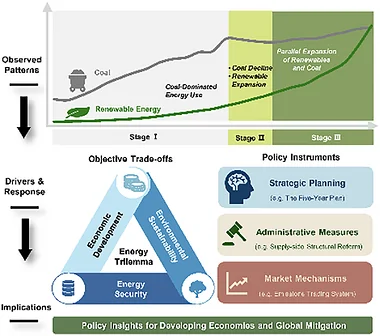

## 1 Introduction

**FIGURE 1:**
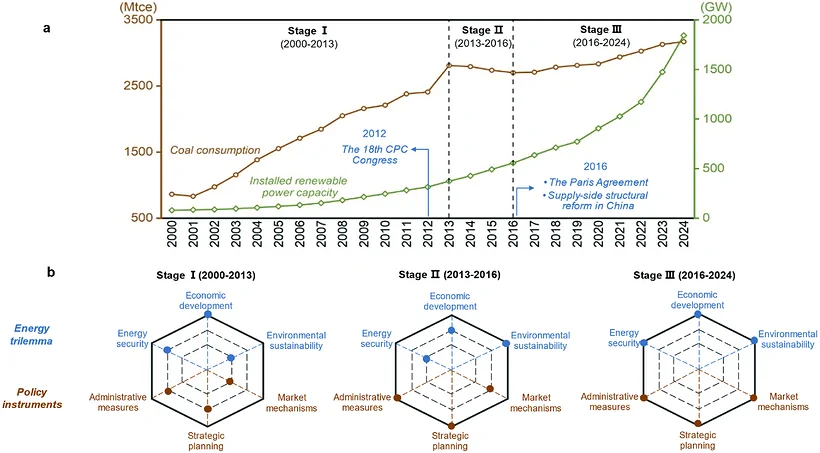
(a) Trends in coal consumption and installed renewable power capacity in China from 2000 to 2024. Coal consumption (left axis, unit: Mtce) increased steadily in the early stage, declined temporarily after 2013, and rebounded from 2016 onward. By contrast, installed renewable power capacity (right axis, unit: GW) expanded continuously throughout the study period. (b) Energy trilemma and policy instruments across different transition stages. The upper half of each radar chart qualitatively represents the relative salience of constraints related to economic development, energy security, and environmental sustainability, while the lower half indicates the relative emphasis placed on strategic planning, administrative measures, and market mechanisms. The radar charts are intended as a qualitative and interpretive summary of stage differences. The position of each point is based on an interpretive judgment derived from the analysis in the text, and the three concentric grid lines indicate low, medium, and high levels, respectively.

As the largest energy consumer and carbon dioxide emitter in the world, China plays a critical role in achieving global climate goals [1, 2]. Unlike most developed economies, where the energy transition followed a path of energy substitution after the completion of industrialization, the transition in China has unfolded alongside ongoing industrialization and urbanization, accompanied by sustained growth in energy demand [3]. It exhibits pronounced stage‐specific characteristics. In the early stages, the expansion of manufacturing and infrastructure construction drove rapid growth in energy demand, with coal maintaining a dominant position due to its cost advantage and domestic resource endowment. Around 2013, under the combined impacts of moderating economic growth and increasing environmental pressures, coal consumption experienced a brief period of decline, while renewable energy began to expand rapidly. However, since 2016, despite the rapid expansion of renewable energy, coal has not undergone a corresponding phase‐out. Instead, it has experienced a significant rebound, forming a pattern of parallel expansion of renewable energy and coal (Figure 1).

Numerous studies have analyzed China's low‐carbon energy transition from different perspectives, with most focusing on the development of the dominant energy at a given stage. For example, during the period of rapid industrialization and urbanization, coal played a crucial role in supporting economic growth and industrial development [4, 5]. The temporary drop in coal consumption around 2013 has been interpreted by some studies as a signal of peaking or even structural decline [6, 7]. Meanwhile, the rapid expansion of renewable energy over the past decade has largely been attributed to technological progress, cost reductions, and policy incentives [8, 9]. While these studies provide strong explanations when applied to specific phases, they pay limited attention to how the energy structure and policy instruments of earlier stages shape the constraints and transition pathways of subsequent periods. On the other hand, some studies have attempted to integrate renewable energy and coal into long‐term analytical frameworks by employing Integrated Assessment Models. However, most studies are based on the assumption of cost minimization, implying that renewable energy will endogenously replace fossil fuels as costs decline [8, 10, 11]. Such studies are effective in identifying cost‐optimal transition pathways in the long term, yet struggle to explain the observed pattern of parallel expansion of renewable energy and coal.

This limitation suggests the need for an analytical perspective that can link different stages of the transition and explain how multiple policy objectives are balanced over time. The energy trilemma provides such a lens. As formulated by the World Energy Council, it emphasizes the need to balance energy security, energy equity, and environmental sustainability, with the equity/affordability dimension often understood in economic terms in the literature [12, 13]. This understanding is particularly important in China, where the energy transition has proceeded alongside industrialization and urbanization, making energy availability and affordability closely tied to macroeconomic growth [14]. Accordingly, this paper operationalizes the energy trilemma as economic development, energy security, and environmental sustainability. Building on this perspective, our study develops an analytical framework that integrates temporal dynamics, energy trilemma, and policy instruments (Figure 1). Based on the macroeconomic context and energy consumption characteristics, the transition process can be divided into three distinct stages. Furthermore, following the classification in the Fourth National Assessment Report on Climate Change of China [15], policy instruments are classified into three categories: strategic planning, administrative measures, and market mechanisms. The analysis compares the relative emphasis placed on each category across stages and examines its contribution to balancing multiple objectives.

This paper makes three main contributions, corresponding to the research perspective, analytical framework, and policy implications. First, from a temporal evolutionary perspective, it traces how policy priorities and energy structure adjustments have evolved across different stages. While existing studies often focus on specific policies, sectors, or stages, this paper highlights the interconnections between stages and explains the transition as a dynamic process. Second, the paper applies the energy trilemma framework to China's low‐carbon energy transition. By focusing on economic development, energy security, and environmental sustainability, it explains how policy priorities have evolved over time and why the rapid expansion of renewable energy has coexisted with a rebound in total coal consumption. Third, the paper identifies how different policy instruments have been combined to address transition constraints. China's experience shows a policy mix in which strategic planning sets the long‐term direction, administrative measures address coordination and implementation challenges, and market mechanisms reshape investment and consumption incentives. These findings provide policy insights for developing economies facing similar transition challenges.

## 2 Long‐Term Coal Dominance in Energy Structure

Before 2013, due to its low cost and abundant resources, coal maintained a dominant position in primary energy consumption and electricity supply, and accounted for nearly 70% of the total energy use. By contrast, renewable energy, mainly hydropower, experienced limited growth under policy support, accounting for less than 10% and serving as a supplementary energy sources.

### 2.1 Energy Growth Driven by Energy‐intensive and High‐emission Industries

After joining the World Trade Organization, China rapidly integrated into global value chains and participated in the international division of labor. Driven by external demand, the manufacturing sector expanded significantly, and China soon became the “Factory of the World”. Existing research indicated that in the early stage, China specialized in primary processing and low value‐added manufacturing, which were typically associated with high energy inputs and emissions [16, 17]. On the domestic demand side, rapid urbanization led to substantial investment in real estate and infrastructure, which drove the expansion of upstream energy‐intensive industries such as steel, chemicals, and building materials, significantly increasing energy consumption [18]. This investment‐driven growth model made capital accumulation a major driver of economic expansion [19]. Propelled by the expansion of both external demand and domestic investment, China's economic growth became increasingly dependent on energy‐intensive and high‐emission industries, leading to a rapid increase in coal consumption.

However, this energy consumption pattern dominated by energy‐intensive and high‐emission industries exhibited significant path dependence and lock‐in effects [20, 21, 22, 23]. Once capital stock, industrial structure, and technological choices were established, the resulting adjustment costs created significant barriers to energy transition. Simultaneously, rising environmental externalities eroded the benefits generated by economic growth, highlighting the unsustainability of this development pattern [24].

### 2.2 Priority of Economic Growth

During this period, economic development was prioritized, and growth relied heavily on energy‐intensive and high‐emission industries. Before 2013, China's gross domestic product (GDP) growth rate once reached over 10% [25]. Industrial output surged from 7.72 trillion CNY in 2005 to 21.07 trillion CNY in 2013, nearly tripling in scale and becoming a key engine of economic growth [26]. The industrial sector accounted for more than 50% of China's final energy consumption, with the five major energy‐intensive industries, including iron and steel, nonmetallic minerals, chemicals, nonferrous metals, and pulp and paper, contributing 73% of industrial energy consumption [27]. Owing to its cost advantage and abundant resource endowment, coal emerged as the primary energy source to meet the rapidly growing energy demand.

In terms of energy security, the primary concern was to alleviate supply shortages and ensure adequate energy availability to support rapid economic growth. In the early 2000s, China experienced widespread power shortages, which significantly disrupted industrial production, with average production costs increasing by approximately 8% [28]. To tackle power shortages and reduce production costs, China initiated power sector reforms in 2002, followed by the large‐scale construction of coal‐fired power plants in subsequent years [29]. It is estimated that from 2004 to 2007, China added an average of 70 GW of coal‐fired power generation capacity annually, equivalent to the total generating capacity of the United Kingdom [30]. Meanwhile, the expansion of coal‐fired power generation stimulated coal production growth, with numerous small coal mines emerging in coal‐producing regions to supplement local supply [31, 32]. This expansion of coal‐based energy infrastructure further reinforced coal dependence.

From the perspective of environmental sustainability, the focus was on enhancing energy efficiency and controlling pollutants, with specific measures primarily centered on reducing energy intensity, tightening emission standards, and promoting end‐of‐pipe treatments such as desulfurization and denitrification. Although carbon mitigation was incorporated into the policy agenda through international climate negotiations, emission reduction targets were mainly expressed as intensity controls rather than constraints on the total level or structure of energy consumption. Consequently, this approach preserved room for the continued expansion of energy consumption [33, 34]. Emission reduction efforts also remained confined to individual sectors or policy objectives, failing to form a governance framework that is coordinated with industrial restructuring and energy transition.

### 2.3 Policy Focus on Energy Conservation and Emission Reduction

During this period, targets for reducing energy intensity as well as major pollutants and carbon dioxide emissions were formally incorporated into national development plans. The 11th Five‐Year Plan set targets to reduce energy consumption per unit of GDP by approximately 20% and total emissions of major pollutants by 10%, explicitly requiring energy consumption per 10 000 CNY of GDP to decrease from 1.22 tce in 2005 to below 1 tce. The 12th Five‐Year Plan further introduced carbon emission reduction, proposing a 17% reduction in carbon emission intensity, echoing China's commitment announced ahead of the 2009 Copenhagen Climate Conference to reduce carbon dioxide emissions per unit of GDP by 40%–45% by 2020 compared to 2005. Regarding renewable energy, the development plan set targets for its share in total energy consumption to reach 10% by 2010 and 15% by 2020 [35], incorporating renewable energy development into national strategic planning.

Administrative measures played a key role in achieving energy conservation and emission reduction at this stage. On the demand side, the “Thousand Enterprises Energy Conservation Action” strengthened energy management in key enterprises, promoted rational energy utilization, and improved energy efficiency [36, 37]. On the supply side, China optimized the structure of coal‐fired power generation by constructing large‐capacity units while retiring nearly 90 GW of small and inefficient units. As a result, generating units with a capacity of 600 MW or above accounted for more than one‐third of total thermal power capacity [38]. Additionally, coal‐fired power plants were required to install desulfurization and denitrification facilities to control end‐of‐pipe pollution [39].

Compared with the immediate impact of administrative measures, market mechanisms remained at the exploratory and pilot stage, with relatively limited influence. The differential electricity pricing policy, implemented in 2006, increased electricity costs for energy‐intensive industries to accelerate the phase‐out of inefficient production capacity [40]. The Feed‐in Tariff mechanism for renewable energy was established in 2009. By setting a fixed feed‐in tariff, the government ensured stable revenue prospects for renewable energy such as wind and solar power, effectively reducing investment risks and driving the expansion of installed capacity [41].

However, the governance approach centered on energy conservation and emission reduction had certain limitations. On the one hand, as energy efficiency continued to improve, the potential for further technical gains narrowed, making incremental improvements increasingly difficult. On the other hand, with increasingly stringent emission standards for major pollutants and the accelerated withdrawal of outdated production capacity, the marginal effectiveness of end‐of‐pipe treatment declined [42]. More importantly, an intensity‐based governance framework was insufficient to fully address total carbon emissions. With the economy maintaining a high growth rate of 7%–10%, total energy consumption and carbon emissions continued to rise despite significant reductions in energy intensity [32].

## 3 Phased Reduction in Coal Consumption

Prior to 2013, economic growth driven by energy‐intensive and high‐emission industries kept coal consumption locked in place under supply security and cost constraints, with emission reductions relying on end‐of‐pipe treatment. As environmental pressures intensified, the policy focus shifted from purely controlling emissions to integrating industrial restructuring and energy transition. Energy‐intensive and high‐emission industries became key targets of supply‐side structural reform, and pollution reduction and carbon mitigation increasingly exhibited synergistic effects. This policy shift created a brief window for coal reduction from 2013 to 2016: coal consumption declined by 2.9% and 3.7% year‐on‐year in 2014 and 2015, respectively [7, 43]. At the same time, renewable energy maintained rapid expansion. By the end of 2016, China's renewable power generation capacity reached 570 GW, constituting 34.6% of total installed power generation capacity [44].

### 3.1 Macroeconomic Transition and Environmental Pressures in Coal Reduction

The 18th National Congress of the Communist Party of China in 2012 marked a phased shift in China's development philosophy, ushering the economy into the “New Normal”. As industrial restructuring progressed, the expansion of energy‐intensive heavy industries slowed markedly, thereby weakening the industrial basis supporting continued growth in coal demand. Tang et al. (2018) found that the phased decline in China's coal consumption stemmed from domestic industrial restructuring and a slowdown in external trade, with industrial restructuring identified as the main internal constraint on coal consumption. Simultaneously, China's embodied coal exports declined significantly after 2011, from 1409 to 1070 Mt in 2015, an average annual decrease of 6.6% [45]. Furthermore, under the dual influence of weakened demand and previous investment, the power sector exhibited overcapacity. Yuan et al. (2016) estimated that by the end of 2015, China's excess installed capacity of coal‐fired power plants reached 80–100 GW; if all approved projects were put into operation, the excess capacity could further expand to approximately 200 GW by 2020, corresponding to stranded assets valued at up to 700 billion CNY [46].

Meanwhile, the environmental externalities resulting from long‐term intensive coal use became acutely visible. In the first quarter of 2013, China experienced severe and persistent haze pollution, affecting an area of approximately 1.3 million square kilometers and a population of about 800 million [47]. In some areas, daily average PM2.5 concentrations exceeded 500 µg m^−^^3^, 20 times the 24‐h World Health Organization air quality guideline [48]. Source apportionment studies revealed that secondary inorganic aerosols constituted an important component of PM2.5, with their key precursors SO_2_ and NO_x_ closely associated with coal burning and power generation [47]. Given these severe health impacts and clear scientific attribution, coal was identified as a primary target of air pollution control, and became a key focus of policy in major regions and industries [49, 50].

Moreover, anti‐dumping and anti‐subsidy investigations targeting Chinese photovoltaic products led to a sharp short‐term decline in China's photovoltaic (PV) exports, falling by 87.5%. The number of new entrants decreased by approximately 10.7%, and the share of exports to Europe plummeted from 67% in 2012 to 30% in 2013 [51, 52]. However, from a long‐term perspective, this external shock accelerated industrial restructuring. The pressure imposed by international trade barriers accelerated the shift of China's PV industry toward the domestic market. To absorb excess production capacity, the government introduced a series of supportive policies, contributing to the rapid expansion of PV installations [52].

### 3.2 Intensified Environmental Constraints and Shifting Trilemma Priorities

During this period, the priority assigned to environmental sustainability increased significantly, economic development shifted from scale expansion to structural optimization, and energy security pressure remained within a controllable range. The relative prioritization of the three objectives experienced a phased adjustment.

In the environmental dimension, China's governance practices gradually shifted from primarily focusing on end‐of‐pipe control to a synergistic approach centered on pollution reduction and carbon mitigation. Shi et al. (2022) found that the synergistic benefits of China's clean air action measures far exceeded the additional carbon dioxide emissions caused by the installation of end‐of‐pipe control equipment, and the net accumulative emission reduction surpassed the accumulated carbon dioxide emission increases in China [53]. During this period, China also actively participated in global climate governance, strengthening its emission mitigation commitments and policy practices in line with international frameworks. International commitments in turn promoted the improvement of the domestic governance system, reflecting a clear pattern of domestic and international synergy [54, 55].

Guided by China's “New Normal” of economic development, greater emphasis was placed on high‐quality development through structural optimization and efficiency improvement. Energy‐intensive and high‐emission industries were no longer the sole drivers of growth. Supply‐side structural reform became a key policy instrument and focused on five priority tasks: cutting overcapacity, reducing excess inventory, deleveraging, lowering costs, and strengthening areas of weakness. As part of the effort to cut overcapacity, China reduced steel production capacity by 45 million tonnes and coal production capacity by 250 million tonnes [56]. In this process, industrial restructuring and energy transition exhibited clear synergies in practice. Guan et al. (2018) pointed out that the structural decline in China's carbon dioxide emissions observed around 2013 resulted from the simultaneous transformation of the industrial and energy systems [57]. Further interprovincial evidence showed that regions advancing both industrial upgrading and energy structure optimization achieved better mitigation performance than those relying on a single adjustment pathway [58].

From an energy security perspective, energy supply conditions remained relatively stable during this period. On the one hand, the coal‐fired power capacity constructed in earlier years gradually came online, coupled with the moderation in economic growth, resulting in a temporary overcapacity in the energy system [59, 60]. On the other hand, installed capacity of wind and solar power expanded rapidly, serving as an important supplement despite not yet being dominant sources of power generation [61, 62]. Under such circumstances, advancing coal reduction and structural adjustment did not impose significant pressure on energy supply security, allowing environmental objectives to assume a dominant role in the short term.

### 3.3 Administrative Measures as the Core Policy Instrument

The Air Pollution Prevention and Control Action Plan was the most representative national strategic planning document during this period. The plan explicitly set targets for controlling coal consumption and implemented a target responsibility system, requiring that coal's share of total energy consumption be reduced to below 65% by 2017. The Beijing‐Tianjin‐Hebei region, the Yangtze River Delta, and the Pearl River Delta were expected to achieve negative growth in total coal consumption. PM2.5 concentrations were targeted to decline by approximately 25%, 20%, and 15%, respectively, while PM10 concentrations in prefecture‐level and above cities were required to decrease by more than 10% compared with 2012 levels [63]. A policy issued by the State Council in the same year established the strategic direction of expanding the domestic photovoltaic market [64].

Compared with strategic planning policies that mainly provided guidance, administrative measures played a more immediate role in the decline of coal consumption during this period. These policies targeted coal combustion sources, including the removal of small coal‐fired boilers, the replacement of residential coal use, and restrictions on highly polluting coal‐based projects [65, 66]. Furthermore, PM2.5 reduction targets were incorporated into the performance evaluation systems of local officials, with some regions even implementing a one‐vote veto system. This compelled local governments to undertake unprecedented enforcement efforts in implementing coal control measures [67].

Market‐based policies created development opportunities for renewable energy through price signals and explored market‐driven pathways for carbon mitigation. In 2013, the National Development and Reform Commission established regional benchmark feed‐in tariffs and a subsidy of 0.42 CNY/kWh for distributed photovoltaic generation, triggering a surge in photovoltaic installations [68]. In the same year, seven pilot carbon emissions trading markets in Beijing, Shanghai, Guangdong, Shenzhen, Hubei, Tianjin, and Chongqing were gradually launched. These pilots effectively promoted greenhouse gas emission reductions of firms in the participating regions and accumulated institutional and governance experience for the establishment of the national carbon market [69, 70, 71].

The period from 2013 to 2016 represented a temporary window for coal consumption decline and served as a critical stage of policy experimentation. During this period, the priority of environmental objectives increased significantly, and environmental governance became a crucial consideration. Through the establishment of strategic targets in national planning, the intensive use of administrative measures, and the initial introduction of market mechanisms, coal consumption declined while the large‐scale expansion of renewable energy progressed. More importantly, this transition exhibited clear characteristics of synergistic governance: pollutant reduction and carbon mitigation advanced in synergy, while industrial restructuring and energy mix optimization complemented each other.

## 4 Parallel Expansion of Coal and Renewable Energy

The decline in coal consumption during the preceding stage occurred under specific circumstances of excess capacity, intensified environmental constraints, and relatively moderate pressure on energy supply. As the macroeconomy gradually stabilized and shifted to a new growth structure, energy demand rebounded. Energy‐intensive and high‐emission industries did not fully exit, and coal chemical industries continued to expand. Emerging sectors such as electric vehicles and data centers grew rapidly, further increasing electricity demand. Meanwhile, constraints related to energy security and industrial supply chain security intensified. Rising geopolitical tensions and the increasing frequency of extreme climate events heightened the requirements for energy system stability. Under conditions of limited power‐system flexibility, further reductions in coal‐fired generation and capacity could have weakened supply reliability. Consequently, after 2016, China's energy transition was characterized by the coexistence of rapid renewable energy expansion and a rebound in coal consumption.

From 2016 to 2024, China's renewable power experienced rapid growth. Newly installed capacity of wind power and solar power ranked first in the world for consecutive years. The cumulative installed capacity of solar power increased from about 77 GW in 2016 to 887 GW by the end of 2024, while wind power capacity rose from about 149 GW in 2016 to 521 GW in 2024 [72]. By the end of 2024, total installed renewable power capacity in China reached 1,889 GW, representing a 25% year‐on‐year increase and accounting for about 56% of total installed capacity, thus occupying a dominant position in the installed capacity structure [73]. While renewable power capacity expanded rapidly, total coal consumption did not decline correspondingly. After 2016, coal consumption reversed its previous decline and began to rise, maintaining strong resilience in the following years, with coal consumption in 2024 exceeding the 2014 level by more than 500 Mtce [74].

### 4.1 Domestic Reform and Global Commitments

In 2016, China's energy transition reached a key turning point as the full launch of supply‐side structural reform coincided with the beginning of the implementation of the Paris Agreement. This historical convergence transformed both domestic priorities for economic stability and international emission mitigation commitments into binding constraints.

Although China's economic growth entered a period of moderation, the large size of its economy continued to exert strong upward pressure on energy demand [75]. Between 2016 and 2024, China's total exports increased from 13.84 trillion CNY to 25.44 trillion CNY [74], and the export structure remained dominated by energy‐intensive products, thereby constituting indirect energy exports [76]. Existing studies indicate that a 1% increase in export volume was associated with an increase of about 0.042% in electricity consumption [77]. At the same time, the rapid development of emerging industries and the progress of electrification significantly increased electricity demand. This trend was also part of a broader global shift, as electricity consumption has increasingly outpaced GDP growth in recent years driven by the expansion of electricity‐intensive activities [78]. In addition, earlier policy adjustments that shifted from dispersed coal use to centralized coal‐fired power generation led to rising electricity demand being satisfied through increased coal consumption [79, 80]. Li et al. (2025) show that the cumulative effect of economic development on coal consumption increment from 2000 to 2022 was 3851.1 Mtce, far exceeding the cumulative effects of other influencing factors [81]. In 2022, the trend magnitude of China's coal consumption increment was 86.09 Mtce, with the production and supply of electric power and heat power contributing 75.76 Mtce [81]. These results suggest that the recent increase in coal consumption has been driven primarily by incremental electricity demand.

Alongside the expansion of demand, there was a growing emphasis on energy security in response to the changes in the external environment. Faced with the reindustrialization strategies promoted by the United States and Europe and the pressure of global industrial chain restructuring, China proposed to ensure a secure supply of key primary commodities and emphasized maintaining a stable share of manufacturing in the economy. Coal thus shouldered the responsibility of ensuring adequate energy supply for the manufacturing industry [82]. To reduce dependence on imported oil and natural gas, the government established four modern coal chemical demonstration zones and promoted projects such as coal to oil, coal to gas, and coal to olefins [83]. These initiatives expanded the role of coal from a single fuel to an important chemical feedstock and created additional demand for coal [84]. An analysis by Centre for Research on Energy and Clean Air (2024) shows that coal consumption in China's coal chemical industry increased by 18% year‐on‐year in the first eight months of 2024 [85]. The expansion of non‐power coal conversion therefore represents an important additional source of coal demand growth.

Meanwhile, although renewable energy capacity expanded rapidly, the development of energy storage and interregional power transmission had not yet fully kept pace. Coal‐fired power still undertook key functions such as frequency regulation and system stability support. Under these conditions, the rebound in coal consumption became a practical response to security constraints and operational requirements [86, 87]. The International Energy Agency (IEA) report on power system flexibility in China highlights that the rapid deployment of wind and solar PV requires greater system flexibility to accommodate increasing shares of variable renewable energy, while current flexibility resources remain insufficient to support large‐scale renewable energy integration [88]. The IEA's Coal 2024 shows that, considering the effects of weather conditions on renewable power generation, China's coal demand could be around 140 Mt higher or lower than the base case during the forecast period, reflecting the uncertainty introduced by fluctuations in renewable electricity output [89]. Coal‐fired power therefore continues to be treated not only as a source of electricity generation, but also as a key resource for grid stability, system flexibility and resource adequacy, which has slowed the pace of coal phase‐out.

Taken together, coal consumption expanded as a stage‐specific outcome of rising electricity demand driven by economic growth, the expansion of non‐power coal conversion, and the continuing need for system support. Incremental electricity demand, driven by exports, manufacturing upgrading, emerging industries, and electrification, remained the most direct source of growth. At the same time, coal chemicals and other conversion sectors sustained feedstock coal demand that does not automatically fall with renewable expansion. Variability in renewable output, uncertainty over hydropower availability, and insufficient storage and interregional transmission capacity further kept coal‐fired power in place as a source of supply adequacy.

The remarkable growth of renewable energy stemmed from cost reductions driven by technological advancements and national development strategies. Over the decade, the total installed capacity of wind and solar photovoltaic power generation increased tenfold, with the unit cost of photovoltaic power decreasing by 73.3% [86]. Scenario simulations based on sustained cost reduction trends indicated that non‐fossil energy generation could account for 62% of total power generation by 2030. Under a renewable‐dominated scenario, the overall cost of the power system would decrease by approximately 11% compared to the baseline scenario [8]. This demonstrated that the large‐scale expansion of renewable energy represented a long‐term rational choice that balanced economic efficiency with emission reduction benefits. At the strategic level, the commitments under the Paris Agreement and China's dual carbon goals provided sustained policy momentum. Studies showed that to meet its commitments under the Paris Agreement, China needed 1500‐2600 GW of wind power capacity and 2200‐2800 GW of solar power capacity by 2050, approximately ten times its 2020 installed capacity [90]. To achieve carbon neutrality, the lock‐in of fossil fuel energy supply systems must be broken. The share of renewable energy in primary energy consumption must increase from approximately 10% in 2020 to nearly 28% by 2035, reaching about 59% by 2050 [91].

### 4.2 Exploration of Multi‐Objective Equilibrium

From 2016 to 2024, China's energy transition entered a phase in which multiple policy objectives imposed tightening constraints simultaneously. The energy system could no longer achieve structural adjustments through the dominance of a single objective. The parallel expansion of coal consumption and renewable energy thus emerged as a phased adaptive model under these conditions.

In terms of economic development, traditional energy‐intensive industries continued to operate, while emerging industries expanded rapidly. On the one hand, steel production remained at a high level for an extended period, cement demand declined after a phase of growth, and basic chemical industries such as petrochemicals and coal chemical industries continued to expand under the joint influence of export demand and domestic consumption [92, 93, 94, 95]. On the other hand, the emergence of the “new trio” (new energy automobiles, lithium batteries, and photovoltaic products) became a key driver of economic growth and industrial upgrading [96]. In 2023, the export value of the “new trio” reached 1.06 trillion CNY, up 29.9% year‐on‐year. Electricity consumption in high‐tech and equipment manufacturing increased by 11.3%, with power use in photovoltaic equipment manufacturing, new energy vehicle manufacturing, and charging and battery‐swapping services rising by 76.8%, 38.8%, and 78.1%, respectively [81]. As digital technologies spread across the economy and society, digital infrastructure such as data centers is also becoming an important source of electricity demand. It is estimated that the ratio of data center electricity consumption to the national total electricity consumption increased from 0.19% in 2006 to 1.70% in 2023 [97]. Traditional industries continue to increase demand for coal and energy‐intensive intermediate goods, while emerging industries are rapidly pushing up electricity demand. Together, these forces have kept coal demand resilient while increasing the need for faster renewable energy deployment.

Regarding energy security, rising uncertainty in the external environment amplified risks. Geopolitical tensions, pandemic disruptions, and extreme weather events increased concerns over energy security, extending the focus from adequate supply to supply chain resilience and power system reliability [98]. In this context, coal plays a foundational safeguarding and system‐support role during the transition. At the same time, coal remains closely linked to the stability of basic industries and manufacturing supply chains. Its supply system is particularly vulnerable to short‐term systemic shocks [99]. Once prices fluctuate, the effects can be transmitted to upstream and downstream industrial chains, thereby amplifying pressures on macroeconomic stability [100, 101]. To reduce such risks, China strengthened coal supply guarantees and price stabilization measures, including the expansion of medium‐ and long‐term coal contracts, which helped maintain stable coal prices and supply conditions. Under these conditions, coal demand expansion reflects not only the need to maintain power system reliability, but also the broader requirement to safeguard industrial supply chains and macroeconomic stability.

From the perspective of environmental sustainability, China's low‐carbon energy transition maintained the synergy between carbon mitigation and pollution control, while shifting its implementation pathway from reducing high‐carbon energy use toward building low‐carbon substitution capacity. Since carbon dioxide and major air pollutants shared a high degree of common origin in energy use and industrial production, carbon emission reduction and pollution control could be synergistically promoted through adjustments to energy and economic structures, comprehensive control of pollution sources, and technological upgrades [102]. The implementation pathway of the transition also changed. In the power sector, it relied more on renewable power expansion than on direct reductions in coal‐fired generation. If coal‐fired generation and capacity are reduced too rapidly before a mature low‐carbon substitution system is in place, the transition could face higher system costs and greater implementation barriers. For this reason, China increasingly emphasized large‐scale expansion of renewable energy to achieve incremental substitution [9]. Environmental sustainability in this stage therefore depended on building renewable‐based substitution capacity first, so that high‐carbon energy could be phased out in a more orderly and less disruptive manner.

### 4.3 Coordinated Policy Instruments for Energy Transition

Strategic planning policies established the long‐term targets of the dual carbon strategy through the “1+N” policy framework, comprising one overarching policy as the core and multiple sector‐specific action plans and supporting policies. This framework brings policies on energy, industry, transport, buildings, and technology into a unified transition agenda, reducing the risk of conflicts caused by fragmented sectoral implementation. It was also systematically aligned with medium‐term policy planning such as the Five‐Year Plans, explicitly proposing a target of achieving total renewable energy consumption of 1 billion tce by 2025 and a non‐fossil energy consumption share of approximately 25% by 2030 [103]. Furthermore, China promoted a shift from energy consumption control to carbon emissions control and newly added renewable energy and energy use for raw materials were excluded from total energy consumption control, thereby creating greater space for renewable energy development [104]. Regarding the pace of transformation, the principle of establishing the new before phasing out the old was clearly proposed, requiring that the withdrawal of traditional energy sources must be premised on the safe and reliable replacement by new energy sources, allowing coal to continue to play a role in ensuring energy system security for a certain period.

Administrative measures focused on transforming the role of coal‐fired power and accelerating the large‐scale expansion of renewable energy. On the one hand, supply‐side reforms in the coal sector deepened to optimize production capacity structures. At the same time, the coordinated program of coal power upgrading, which included improvements in energy efficiency, heating supply, and operational flexibility, was widely implemented to facilitate the transition of coal‐fired power from a primary electricity source to a flexible power source [105]. During the 14th Five‐Year Plan period, the program targeted energy‐efficiency retrofits for around 350 GW of coal‐fired power units, heating‐supply retrofits for around 50 GW, and flexibility retrofits for around 200 GW. On the other hand, in the renewable energy sector, the government accelerated the development of large‐scale wind and solar power bases. Resource‐rich regions in western and northern China were promoted as major low‐carbon power supply hubs, serving load centers in central and eastern China through ultra‐high‐voltage transmission and other interregional delivery channels. Through administrative coordination of land resources, transmission infrastructure, and regional electricity consumption obligations, these policies created the institutional and infrastructural foundations for integrating a high share of renewable energy into the power system [106, 107].

Market‐based policy instruments aimed to establish a multidimensional price signal system to guide energy development toward market‐driven dynamics. On the one hand, the policy of grid parity for renewable energy was implemented, and national fiscal subsidies were gradually phased out, forcing renewable power to compete on equal footing with coal‐fired power through cost reductions achieved by technological advancements [108]. On the other hand, the capacity‐based pricing mechanism for coal‐fired power also reshaped the revenue structure of coal plants by compensating capacity availability and system‐support services. This allowed some units to reduce generation while retaining their reliability functions and to withdraw from regular operation without being dismantled [109]. At the same time, the accelerated development of the national unified electricity market, the national emissions trading system, and the China Certified Emission Reduction (CCER) market together formed a market framework for the energy transition [110, 111]. Electricity market reform has strengthened price signals in China's electricity system by linking medium‐ and long‐term trading with spot and ancillary service markets. This allows electricity prices to better reflect supply‐demand conditions [112]. The national emissions trading system incorporates emission costs into firms’ operational decisions, imposing more explicit carbon constraints on high‐carbon generating units and high‐emission firms [113]. The restart of the CCER market further provides a complementary channel for emission‐reduction revenues from projects such as forestry carbon sinks, renewable energy, and methane abatement [114]. Together, these mechanisms strengthen electricity price and carbon price signals, guiding the gradual exit of high‐carbon assets and incentivizing investment in low‐carbon technologies.

The coexistence of rapid renewable energy expansion and a rebound in coal consumption does not reflect a deviation from or reversal of China's energy transition. Rather, it represents a temporary equilibrium that emerged under the simultaneous tightening of constraints related to economic development, energy security, and environmental sustainability. The principle of establishing the new before phasing out the old, while maintaining energy security, has secured a crucial opportunity for the large‐scale expansion of renewable energy and a rapid reduction in costs. At the same time, the role of the market in resource allocation continues to strengthen: electricity markets, capacity compensation mechanisms, green power trading, and carbon markets are gradually transitioning from pilot programs to systematic operation, laying the market foundation for a smooth transition toward a clean energy‐dominated future.

## 5 Policy Insights and Outlook

### 5.1 Policy Lessons from China's Low‐Carbon Energy Transition

This study summarizes China's key governance experiences in the low‐carbon energy transition from a long‐term evolutionary perspective, which are mainly reflected in the aspects of objective trade‐offs, policy arrangements, pathway choices, and governance orientation.

Regarding objective trade‐offs, the prioritization of the energy trilemma exhibits a clear temporal evolution across different development stages. In the early stages, economic development served as the core objective, while environmental performance was improved through energy conservation, efficiency enhancement, and end‐of‐pipe treatment under the premise of ensuring energy supply. As environmental pressures intensified and capacity reduction policies progressed, carbon mitigation and pollution control were temporarily prioritized. When the constraints of economic development, energy security, and environmental sustainability tightened simultaneously, policy priorities shifted toward enhancing compatibility among these objectives through institutional design. The key is to reduce direct conflicts among policy objectives by adjusting the sequencing and arrangements of policy instruments across different stages.

In terms of policy arrangements, China's low‐carbon energy transition has gradually developed a multidimensional policy framework involving strategic planning, administrative measures, and market mechanisms. Strategic planning policies define medium‐ and long‐term development objectives and serve as the primary instruments for setting the strategic direction of the transition. Administrative measures play a leading role in policy implementation and are effective in coordinating the actions of different actors in the short term. As the transition moves into a deeper stage, market mechanisms are gradually strengthened. Through energy prices and social carbon costs that more fully reflect resource scarcity and environmental externalities, the market drives investment and consumption decisions toward low‐carbon pathways.

In pathway choice, China has adopted an incremental transition model characterized by the principle of establishing the new before phasing out the old. On the one hand, the country has continuously expanded the large‐scale deployment of renewable energy sources such as wind and solar power, while simultaneously promoting the development of system infrastructure including power grids and energy storage, thereby creating the conditions for progressive low‐carbon substitution. On the other hand, coal‐fired power has undergone functional transformation rather than a simple phase‐out, allowing it to maintain roles related to system stability and reliability. This arrangement avoids supply imbalances and cost shocks that could arise from an overly rapid retirement of dispatchable coal‐fired capacity.

In governance orientation, the low‐carbon energy transition has gradually shifted from a single‐objective approach toward a coordinated and integrated governance framework. Early governance focused on controlling conventional air pollutants, but as carbon constraints strengthened, it gradually evolved toward the coordinated reduction of air pollutants and carbon emissions. Long‐term mitigation objectives are translated into phased targets within Five‐Year Plans and specialized action programs, aligning long‐term strategic goals with medium‐term policy implementation. Energy transition has been closely integrated with industrial restructuring, as adjustments in industrial structure reshape energy demand patterns, while the development of low‐carbon energy supports industrial upgrading. Regional coordination has also become an important feature of the transition, with renewable energy development in resource‐rich western and northern regions supporting power supply for major demand centers and creating new opportunities for local economic development.

### 5.2 Implications for Developing Countries and Global Energy Transition

Developing countries generally face the real pressure of simultaneously advancing economic development, energy security, and environmental sustainability targets before industrialization is complete. On the one hand, industrialization and urbanization still require a stable and affordable energy supply; on the other hand, global climate governance and green trade rules require these countries to enter the low‐carbon transition earlier. The historical pathway followed by developed economies in which industrialization preceded decarbonization is difficult to replicate, and excessively rapid reduction in fossil fuel consumption may lead to energy supply fluctuations in the short term. Therefore, for developing countries, the key to a low‐carbon transition is how to arrange a reasonable sequence.

The reference value of China's experience lies primarily in its governance approach under multiple constraints. Low‐carbon energy transition needs to be aligned with the stage of development, adjusting the relative priorities of development, security, and mitigation. Before clean energy forms a stable alternative, a too‐rapid withdrawal of traditional energy sources can amplify supply risks. Policy implementation also requires a combination of tools: planning to determine direction, administrative measures to resolve infrastructure and inter‐departmental cooperation issues, and market mechanisms to gradually change investment and consumption choices.

The Chinese approach also has clearly defined boundaries of application. The large‐scale expansion of renewable energy, the flexible transformation of coal‐fired power plants, and the continued implementation of policy objectives rely on China's massive market, complete manufacturing system, and policy execution capabilities. Many developing countries may have shortcomings in such areas. Ignoring these differences and directly copying China's policy pace or governance methods may increase investment pressure and systemic operational risks. China's policy implementation has also evolved by accounting for regional conditions and industrial characteristics, gradually expanding from targeted interventions in key regions and sectors to broader economy‐wide governance. Therefore, specific transition strategies need to be tailored to each country's resource endowment, industrial structure, and governance capabilities.

These unique and difficult‐to‐replicate conditions further highlight the practical significance of international cooperation. Many developing countries face constraints in clean energy supply chains, technological capabilities, and market mechanisms. Relying solely on domestic resources to drive transition often faces high costs. International cooperation can help address these constraints by facilitating technology transfer, knowledge sharing, and financial support. Developed countries possess advantages in advanced low‐carbon technologies, while multinational enterprises can contribute through cross‐border investment and technological cooperation. Meanwhile, emerging economies with expanding clean energy markets can create new opportunities for international collaboration. By leveraging the comparative advantages of different countries, international cooperation can accelerate the low‐carbon energy transition and create more feasible pathways toward global climate goals.

### 5.3 Future Outlook

Looking ahead, China's low‐carbon energy transition will be shaped by the coordinated evolution of economic development, energy security, and environmental sustainability.

From the perspective of economic development, the coexistence of traditional and emerging industries will remain for a considerable period. Under the framework of the 15th Five‐Year Plan, traditional industries such as mining, metallurgy, chemicals, building materials, and machinery will continue to play an important role in stabilizing employment and investment, while maintaining China's position in the global division of labor. These traditional industries are expected to pursue quality upgrading and structural transformation through technological renovation, digital transformation, and green manufacturing instead of shrinking in scale. At the same time, emerging and future industries such as new energy, aerospace, and the low‐altitude economy are positioned as new pillars of economic growth. As a result, future energy demand will increasingly arise from the rapid expansion of emerging industries.

Regarding energy security, future energy system stability will increasingly depend on the coordinated advancement of structural adjustments and system construction. On the one hand, the focus of energy security is gradually shifting from ensuring fossil fuel supply toward building a supply system centered on renewable energy. According to China's latest Nationally Determined Contributions (NDCs), the share of non‐fossil energy consumption is projected to exceed 30% by 2035, while the installed capacity of wind and solar power will reach approximately 3600 GW. This implies that incremental electricity demand will be met by renewable generation. Coal‐fired power is expected to undergo a functional transformation through efficiency improvements, flexibility retrofits, and the orderly retirement of less efficient units. On the other hand, under conditions of high shares of renewable energy, energy security will increasingly depend on the operational resilience of the energy system. This requires coordinating local consumption with interregional transmission, accelerating the deployment of pumped storage and new energy storage technologies, and promoting the construction of smart grids to provide reliable support for the low‐carbon energy transition.

In terms of environmental sustainability, China's low‐carbon energy transition will increasingly emphasize the coordination between environmental quality improvement and carbon mitigation. Under the goal of building a Beautiful China by 2035, environmental standards and pollution control requirements will continue to strengthen, creating further pressure for regions and industries to improve environmental quality. At the same time, China's updated NDCs introduce additional constraints on greenhouse gas emissions, with the goal of reducing economy‐wide net greenhouse gas emissions by 7%–10% from their peak level by 2035 through adjustments in spatial patterns, industrial structure, transportation systems, and the energy mix. Together, these objectives suggest that future energy transition pathways will need to enhance synergies between pollution reduction and carbon mitigation, further reinforcing the importance of coordinated governance.

The pursuit of multiple objectives implies that China's low‐carbon energy transition will face increasingly complex and interconnected challenges in the future. First, the rapid development of emerging industries and the associated growth in electricity demand will continue to place pressure on the energy system for a considerable period. Second, while the share of renewable energy continues to rise rapidly, there remain potential risks of lagging development in system flexibility, interregional coordination capacity, and the availability of regulatory resources. In addition, the transition from coal‐based energy systems toward renewable energy will proceed at a different paces across regions and industries, as their existing energy structures and development conditions differ, leading to diverse transition pathways and uncertainty in the process.

Building on the long‐term evolutionary characteristics of China's low‐carbon energy transition, future research can be advanced along three main directions. First, research should move beyond stylized assumptions of price‐based substitution and better identify how policy interventions shape the development trajectories of coal and renewable energy. Future studies should further develop dynamic evaluation frameworks incorporating policy sequencing to examine how the prioritization of objectives across stages influences long‐term transition pathways and costs. Second, research should identify the timeline for phasing out coal power and examine the conditions required for renewable power to become the dominant source of electricity without locking in new coal capacity. Finally, attention should be paid to technological breakthroughs in energy storage, carbon capture, utilization, and storage, as well as the growing energy demands from emerging industries such as artificial intelligence and data centers, to assess how technological progress and evolving demand will influence the pace of the transition.

## Author Contributions

**Yu Liu**: conceptualization, funding acquisition, methodology, Writing – review and editing, project administration, resources. **Zhiyu Tian**: conceptualization, investigation, methodology, writing – review and editing, supervision, resources. **Zhengxian Zhang**: conceptualization, investigation, writing – original draft, validation, visualization, formal analysis, data curation, writing – review and editing, software. **Xinbei Li**: writing – original draft, visualization, writing – review and editing, software, supervision.

## Funding

This work is supported by the National Natural Science Foundation of China (No. 72125010, No. 72561147303), the Postdoctoral Fellowship Program of China Postdoctoral Science Foundation (No. GZC20261046), and the Fundamental Research Funds for the Central Universities, High‐performance Computing Platform of Peking University.

## Conflicts of Interest

The authors declare no conflicts of interest.

## Data Availability

The data that support the findings of this study are available from the corresponding author upon reasonable request.
